# Point-of-care molecular diagnosis of *Mycoplasma pneumoniae* including macrolide sensitivity using quenching probe polymerase chain reaction

**DOI:** 10.1371/journal.pone.0258694

**Published:** 2021-10-14

**Authors:** Nobuhisa Ishiguro, Rikako Sato, Toshihiko Mori, Hiroshi Tanaka, Mitsuo Narita, Takashi Nagano, Masato Owaku, Kensuke Miyajima, Atsushi Manabe

**Affiliations:** 1 Department of Pediatrics, Hokkaido University Graduate School of Medicine, Sapporo, Hokkaido, Japan; 2 Department of Pediatrics, NTT East Sapporo Hospital, Sapporo, Hokkaido, Japan; 3 Sapporo Cough Asthma and Allergy Center, Sapporo, Hokkaido, Japan; 4 Department of Pediatrics, Sapporo Tokushukai Hospital, Sapporo, Hokkaido, Japan; 5 MIZUHO MEDY Co., Ltd., Tosu, Japan; University of Helsinki: Helsingin Yliopisto, FINLAND

## Abstract

**Objectives:**

Macrolides are generally considered to be the drugs of choice for treatment of patients with *Mycoplasma pneumoniae* infection. However, macrolide-resistant *M*. *pneumoniae* has been emerging since about 2000. The Smart Gene^®^ system (MIZUHO MEDY Co., Ltd., Tosu, Japan) is a novel fully automated system for detection of pathogens using the method of quantitative polymerase chain reaction (qPCR) with QProbe (QProbe PCR). The entire procedure is completed within 50 min and the size of the instrument is small (15 x 34 x 30 cm). The purpose of this study was to evaluate the usefulness of the Smart Gene^®^ system for detection of *M*. *pneumoniae* and detection of a point mutation at domain V of the 23S rRNA gene of *M*. *pneumoniae*.

**Materials:**

Pharyngeal swab samples were collected from 154 patients who were suspected of having respiratory tract infections associated with *M*. *pneumoniae*.

**Results:**

Compared with the results of qPCR, the sensitivity and specificity of the Smart Gene^®^ system were 98.7% (78/79) and 100.0% (75/75), respectively. A point mutation at domain V of the 23S rRNA gene was detected from 7 (9.0%) of 78 *M*. *pneumoniae*-positive samples by the Smart Gene^®^ system and these results were confirmed by direct sequencing. The minimum inhibitory concentrations of clarithromycin among the 5 isolates of *M*. *pneumoniae* with a point mutation at domain V of the 23S rRNA gene were >64 μg/ml and those among the 33 isolates without a mutation in the 23S rRNA gene were <0.0625 μg/ml.

**Conclusion:**

The Smart Gene^®^ system is a rapid and accurate assay for detection of the existence of *M*. *pneumoniae* and a point mutation at domain V of the 23S rRNA gene of *M*. *pneumoniae* at the same time. The Smart Gene^®^ system is suitable for point-of-care testing in both hospital and outpatient settings.

## Introduction

*Mycoplasma pneumoniae* (*M*. *pneumoniae*) is one of the common causative pathogens of community-acquired respiratory tract infections in humans, especially in children and young adults [1]. It was reported that *M*. *pneumoniae* accounted for a steadily increasing proportion of cases of pneumonia with increasing age of the children: 2% (<2 years), 5% (2–4 years), 16% (5–9 years) and 23% (1–17 years) [2]. Macrolides are generally considered to be the drugs of choice for treatment of patients with *M*. *pneumoniae* infection [3]. However, macrolide-resistant *M*. *pneumoniae* (MRMP) has been emerging in Asia, Europe, Canada and the USA since about 2000 [4–7]. The Macrolide resistance rates were 0% to 15% in Europe and the USA, approximately 30% in Israel and up to 90%-100% in Asia [8]. Minocycline and fluoroquinolones were shown to be more effective than macrolides in adult patients infected with MRMP [9]. Minocycline and tosufloxacin have also been used for treatment of patients infected with MRMP [10–13]. Tetracyclines including minocycline are incorporated into teeth, cartilage and bone, resulting in discoloration of both primary and permanent dentitions [14, 15]. Therefore, tetracyclines are contraindicated in children aged less than 8 years [14, 15]. Fluoroquinolones including tosufloxacin have a potential risk of inducing cartilage and joint toxicity in children [15]. The guidelines for the treatment of respiratory infectious diseases by the Japanese Association for Infectious Diseases (JAID) and Japanese Society of Chemotherapy (JSC) recommend the use of tosufloxacin or tetracycline for pediatric patients diagnosed with MRMP pneumonia [16]; however, the clinical effects of tosufloxacin in pediatric patients infected with MRMP have been controversial [13].

Antimicrobial resistance (AMR) is a global public health concern and unnecessary use of antibiotics has contributed to the global emergence of antimicrobial resistance [17]. Rapid and accurate diagnostic techniques for identifying the causative pathogen would be useful for initiating treatment with an appropriate antibiotic from the perspective of AMR. Nucleic acid amplification techniques (NAATs) including quantitative polymerase chain reaction (qPCR) assay, multiplex qPCR assay and loop-mediated isothermal amplification (LAMP) have been increasingly used for identification of respiratory pathogens including *M*. *pneumoniae* in clinical specimens due to their high levels of sensitivity and specificity [18, 19]. QPCR and multiplex qPCR assays usually require high-performance instruments and well-equipped laboratories, and these assays can detect macrolide-resistant point mutations at domain V of the 23S rRNA gene of *M*. *pneumoniae*. The instrument used for LAMP is relatively inexpensive but macrolide-resistant point mutations at domain V of the 23S rRNA gene of *M*. *pneumoniae* cannot be detected by LAMP [20–23]. Recently, a portable system for 16S rRNA analyses consisting of a nanopore technology-based sequencer (MinION, Oxford Nanopore Technologies, Oxford, UK) has been developed and has been used for research and diagnosis of infectious diseases [24]. This novel technology could be used for diagnosis of infection caused by *M*. *pneumoniae*; however, knowledge and skills of molecular biology are required.

The Smart Gene^®^ system (MIZUHO MEDY Co., Ltd., Tosu, Japan) was developed on the basis of the concept of point-of-care testing for genetic testing of pathogens. This system does not require any special technique. The Smart Gene^®^ system automatically performs nucleic acid extraction and gene detection using the method of qPCR with QProbe (QProbe PCR), and the use of the system was approved in Japan in 2018 [25–27]. The Smart Gene^®^ system consists of a fully automatic gene analyzer (Smart Gene^®^) and a dedicated cartridge (Smart Gene^®^ Myco). All of the reagents necessary for extraction of nucleic acids, amplification and detection of the targeted sequences of *M*. *pneumoniae* are included in the dedicated cartridge (Smart Gene^®^ Myco) [28]. After setting the dedicated cartridge (Smart Gene^®^ Myco) into the gene analyzer (Smart Gene^®^), nucleic acids are extracted from samples by a silica solid phase extraction method [29, 30] and the genomic region encoding 23S ribosomal RNA (23S rRNA) is amplified by QProbe PCR. The existence of the genome of *M*. *pneumoniae* and point mutations at domain V of the 23S rRNA gene of *M*. *pneumoniae* are analyzed simultaneously with melting curve analysis using the quenching probe method [26, 27]. A macrolide inhibits protein synthesis mainly by binding to domain V of the 23S rRNA at nucleotide positions 2063 and 2064; these positions appear to be essential for binding [31, 32]. Therefore, mutations at A2063 or A2064 confer the highest resistance to these antimicrobials [31]. The entire operation from setting of the cartridge to obtaining the result proceeds automatically and the entire procedure is completed within 50 min. Contamination due to handling should be minimal. The gene analyzer (Smart Gene^®^) is small (152 mm (W), 343 mm (D) and 300 mm (H)) and inexpensive ($5,000).

It has been reported that NAATs were used in only 3.8% of patients who were suspected of having *M*. *pneumoniae* infection in Japan [33]. For the majority of patients, antibody testing (65.6% of patients) and antigen testing including an immunochromatographic assay (30.6% of patients) were used [33]. The Smart Gene^®^ system is expected to be useful in a medical setting for point-of-care testing, especially in countries such as Japan in which NAATs are not widely used.

The purposes of this study were (1) to determine the sensitivity and specificity of the Smart Gene^®^ system for detection of *M*. *pneumoniae* from pharyngeal swab samples compared with the results of qPCR, (2) to confirm the results for point mutations at domain V of the 23S rRNA gene of *M*. *pneumoniae* detectable in the Smart Gene^®^ system by direct sequencing and (3) to confirm the *in vitro* anti-mycoplasma activities of antibiotics against isolates of *M*. *pneumoniae* with or without a point mutation at domain V of the 23S rRNA gene detected by the Smart Gene^®^ system.

## Materials and methods

### Ethical approval

Ethical approval for this study was obtained from the Institutional Review Board of Hokkaido University Hospital for Clinical Research (018–0051).

### Study population and procedures

Pharyngeal swab samples were collected from patients who were suspected of having respiratory tract infections associated with *M*. *pneumoniae* from July 1, 2018 to December 31, 2019 at the Sapporo Cough, Asthma, and Allergy Center and the Department of Pediatrics in NTT-East Sapporo Hospital, Sapporo, Japan. Two swabs were used for collection of specimens: one for QProbe PCR and the other for isolation of *M*. *pneumoniae*. A swab provided with the Smart Gene^®^ Myco was slowly inserted from the oral cavity into the pharynx, and mucous membrane was collected by rubbing the posterior pharyngeal wall or faucial tonsil several times. The other swab for isolation of *M*. *pnuemoniae* was suspended in Universal Transport Medium (UTM, Copan Diagnotics Inc, Italy) and then stored in the deep freezer.

The age and sex of each patient, the time of onset (the first time that the patient had a fever of more than 37.5˚C), symptoms of the respiratory system (cough, nasal discharge, sore throat, wheezing, dyspnea, headache and fatigue), radiographic findings, antibiotics taken within one week if any and diagnosis (pneumonitis, bronchitis or upper respiratory tract infection) were recorded by the physicians. Written informed consent was obtained from all patients or guardians.

### Detection of *M*. *pneumoniae* with the Smart Gene^®^ system

Smart Gene^®^ Myco is a cartridge in which nucleic acid extraction and QProbe PCR are carried out. The tip of a swab containing a specimen is dipped into the extraction reagent containing guanidine thiocyanate and Triton-X100, and the nucleic acids are extracted from the specimen. Four drops of the extraction reagent containing nucleic acids are placed onto the sample spot of the cartridge (S1 Fig). The nucleic acids in the sample are trapped on the surface of the silica particles in the membrane filter. After washing with the washing buffer, the nucleic acids on silica particles are used for the following QProbe PCR reaction (S2 Fig).

**Fig 1:**
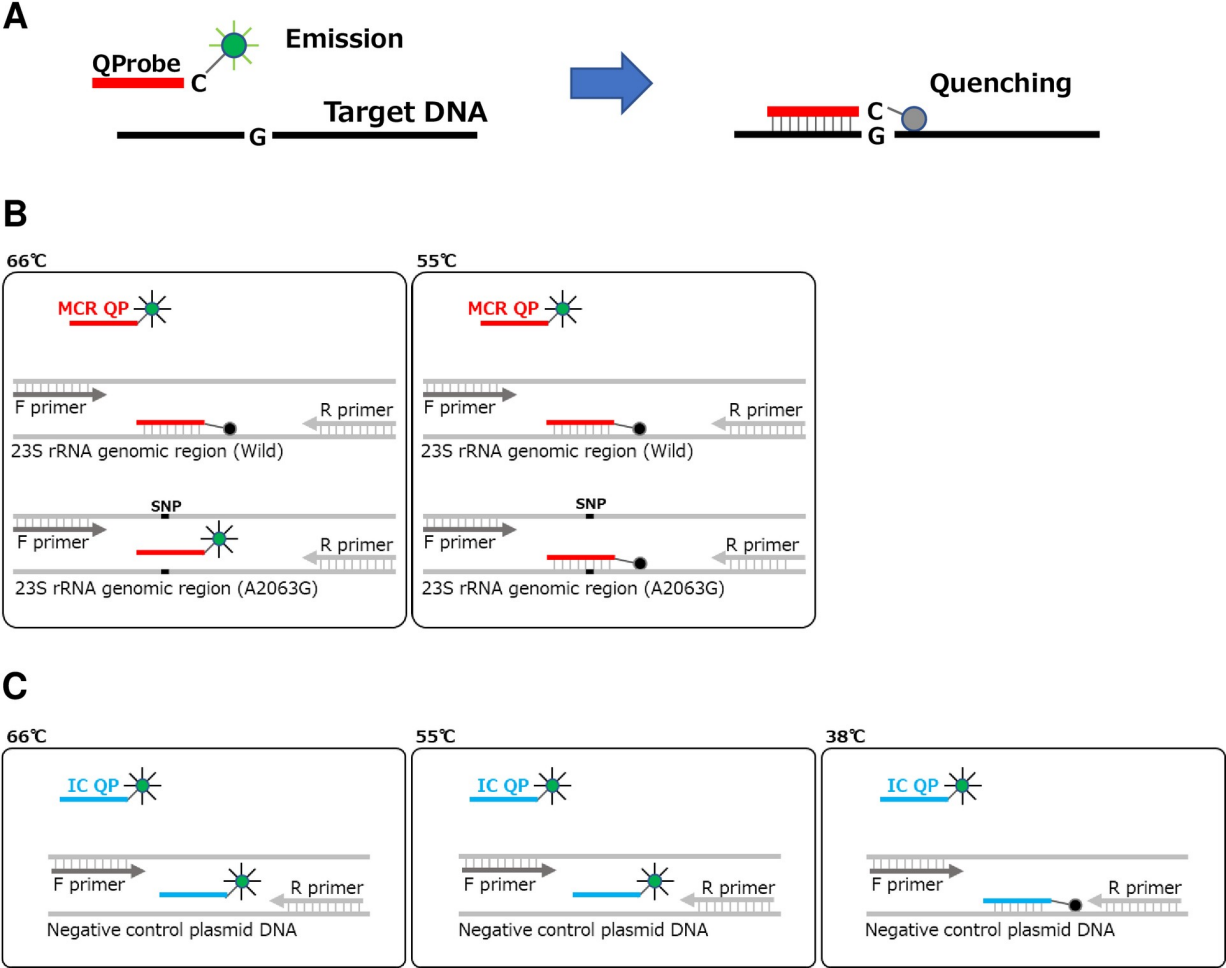
Schematic diagram of the PCR method using QProbe (A) and relationship between temperature and fluorescence intensity in measurements of samples (B) and in tests of internal controls (C).

The Smart Gene^®^ system uses the PCR method with QProbe [26, 27]. QProbe is a hybridization probe in which the terminal cytosine is labeled with a fluorescent dye that quenches upon binding to the target sequence, and it is able to differentiate single-nucleotide polymorphisms using melting curve analysis [26, 27]. QProbe emits upon dissociation to the target sequence (Fig 1A, left) and quenches upon binding to the target sequence (Fig 1A, right). Resistance to macrolides results from a mutation at any one of the positions 2063, 2064, 2067 and 2617 in domain V of the 23S rRNA gene within the mycoplasma cell, which causes a subsequent conformational change and precludes macrolide binding [4, 34]. The specific primers of PCR were designed to amplify the genomic region of domain V of the 23S rRNA gene containing positions 2063, 2064 and 2067 at domain V of the 23S rRNA gene. The specific QProbe for *M*. *pneumoniae* (MCR QP) was designed to bind to the sequences containing positions 2063, 2064 and 2067 at domain V of the 23S rRNA gene, and the sequences of MCR QP are identical to those of the wild-type strain. The melting temperature (Tm) of the specific primers and the MCR QP are set to around 66°C. If the genome sequence of *M*. *pneumoniae* is homologous to the wild type, the MCR QP anneals to the PCR product and QProbe fluorescence is quenched at either 55°C or 66°C (Fig 1B). If the genome sequence of *M*. *pneumoniae* has a mutation at any one of the positions 2063, 2064 and 2067 in domain V of 23S rRNA, the MCR QP does not anneal to the PCR product at 66°C and QProbe fluorescence is quenched at 55°C but not at 66°C (Fig 1B). If the genome sequence of *M*. *pneumoniae* is not detected, the MCR QP does not anneal to the PCR product and QProbe fluorescence is not quenched at either 55°C or 66°C.

**Fig 2:**
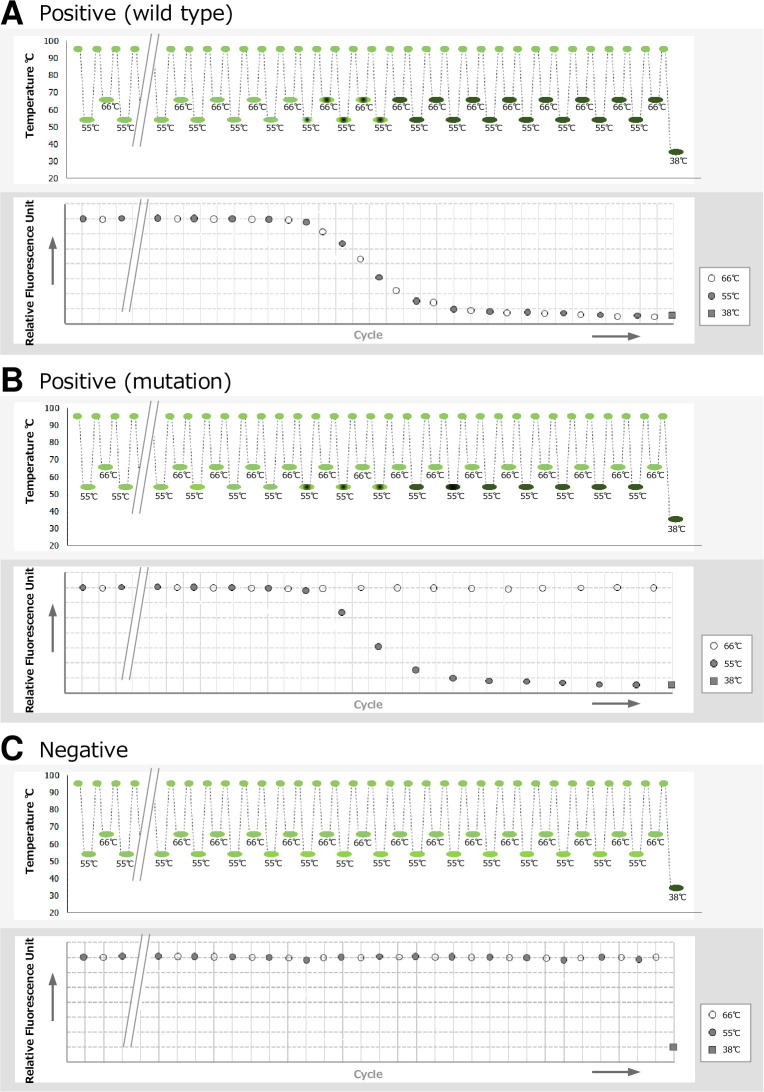
Schematic diagram of the amplification curve using a sample containing wild-type *M*. *pneumoniae* (A), a sample containing mutant-type *M*. *pneumoniae* (B) and a sample without *M*. *pneumoniae* (C). In the graph showing temperature cycling, green ellipsoids indicate the emission of QProbe and black ellipsoids indicate the quenching of QProbe. In the amplification curve, the fluorescence intensity of reactions is shown at 66°C (white circle), 55°C (black circle) and 38°C (black square).

The PCR protocol involves denaturation at 98°C for 120 sec followed by 46 cycles of annealing and extension at 55°C or 66°C for 20 sec and denaturation at 95°C for 10 sec. The results of the Smart Gene^®^ system are displayed as follows. (1) If the genome sequence of *M*. *pneumoniae* is identical to the wild type, the MCR QP anneals to the PCR product and QProbe fluorescence is quenched at either 55°C or 66°C (Figs 1B and 2A). The Smart Gene^®^ system reports “MP-positive and no mutation”. (2) If the genome sequence of *M*. *pneumoniae* has a mutation at any one of the positions 2063, 2064 and 2067 in domain V of the 23S rRNA gene, the MCR QP does not anneal to the PCR product at 66°C and QProbe fluorescence is quenched at 55°C but not at 66°C (Figs 1B and 2B). The Smart Gene^®^ system reports “MP-positive and mutation”. (3) If the genome sequence of *M*. *pneumoniae* is not detected, the MCR QP does not anneal to the PCR product and QProbe fluorescence is not quenched at either 55°C or 66°C (Fig 2C). The Smart Gene^®^ system reports “MP-negative”.

The Smart Gene^®^ Myco includes a negative control plasmid that contains identical sequences to the specific primers of *M*. *pneumoniae* but does not contain sequences homologous to the MCR QP. The internal control QProbe (IC QP) was designed to bind to the intervening sequences between annealing sites of the specific primers of *M*. *pneumoniae* in the negative control plasmid. Because the Tm of IC QP is set to around 38°C, the IC QP does not anneal to the PCR product and QProbe fluorescence is not quenched during the PCR test (The temperature of the rection mixture is over 55°C during the PCR reaction.). After completion of the PCR reaction, the temperature of rection mixture declines to 38°C, the control QProbe anneals to the PCR product and QProbe fluorescence is quenched (Fig 1C). If QProbe fluorescence is quenched at 38°C along with the decrease in the temperature of the rection mixture, the PCR reaction is shown to be successful (Fig 2C). The positive control plasmid for the Smart Gene^®^ Myco is prepared separately. Because the Smart Gene^®^ system uses one reaction tube per sample, the PCR reaction cannot be verified by the positive control plasmid in one reaction tube. The PCR reaction should be verified by the positive control plasmid in another reaction tube.

### Detection of *M*. *pneumoniae* with qPCR

In order to evaluate the sensitivity and specificity of the Smart Gene^®^ system for detection of *M*. *pneumoniae* in comparison with those of qPCR, DNA was extracted with a DNA extraction kit (SMITEST EX-R&D, Medical & Biological Laboratories Co., Nagoya, Japan) from 100 μL of the extraction reagent solution and was finally resuspended in 15 μL of the buffer. One μL of DNA solution was quantified by qPCR using Mp181-F and Mp181-R primers and an Mp181-P probe as described elsewhere [13, 35, 36].

### Detection of macrolide-resistant point mutations at domain V of the 23S rRNA gene

In order to determine whether the Smart Gene^®^ system can detect macrolide-resistant strains of *M*. *pneumoniae*, mutations associated with resistance to macrolides at sites 2063, 2064, 2067 and 2617 in domain V of the 23S rRNA gene in *M*. *pneumoniae* were detected by a sequencing method described elsewhere [37]. *M*. *pneumoniae* possessing a point mutation at domain V of the 23S rRNA gene was defined as MRMP.

### Isolation of *M*. *pneumoniae* and determination of minimum inhibitory concentrations (MICs)

Isolation of M. pneumoniae and determination of MICs were performed according to a previous report [34]. Modified Hayflick medium was used for the isolation of *M*. *pneumoniae* from patients. MICs of clarithromycin (CAM), Minocycline (MINO) and Tosufloxacin (TFLX) were determined by a broth microdilution method using a 96-well dry plate (Eiken Chemical Co., Tokyo, Japan). The broth medium containing 2 x 10^4^ to 3 x 10^5^ CFU/ml of *M*. *pneumoniae* was placed in a 96-well dry plate prepared with serial twofold dilutions of antibiotics. The dry plates were incubated at 37°C for up to 18 days (median: 7 days, interquartile range: 6–8 days). The MICs were determined as the lowest concentrations of antibiotics that inhibited visible growth of *M*. *pneumoniae*. Examples of MIC determination using MRMP and macrolide-sensitive *M*. *pneumoniae* (MSMP) strains are shown in S5 Fig.

### Statistical analyses

The sensitivity, specificity, positive predictive value, and negative predictive value of Smart Gene^®^ Myco were calculated using the Clopper and Pearson method with 95% confidence intervals (CIs). Standard deviation at age was calculated using the Wilcoxon rank sum test. Categorical variables were compared by Fisher’s exact test. A p-value of less than 0.05 was considered statistically significant. All calculations were conducted using the R 4.1.0 software program (The R Foundation, Vienna, Austria).

## Results

### Patient characteristics and detection of *M*. *pneumoniae* by qPCR

**Fig 3:**
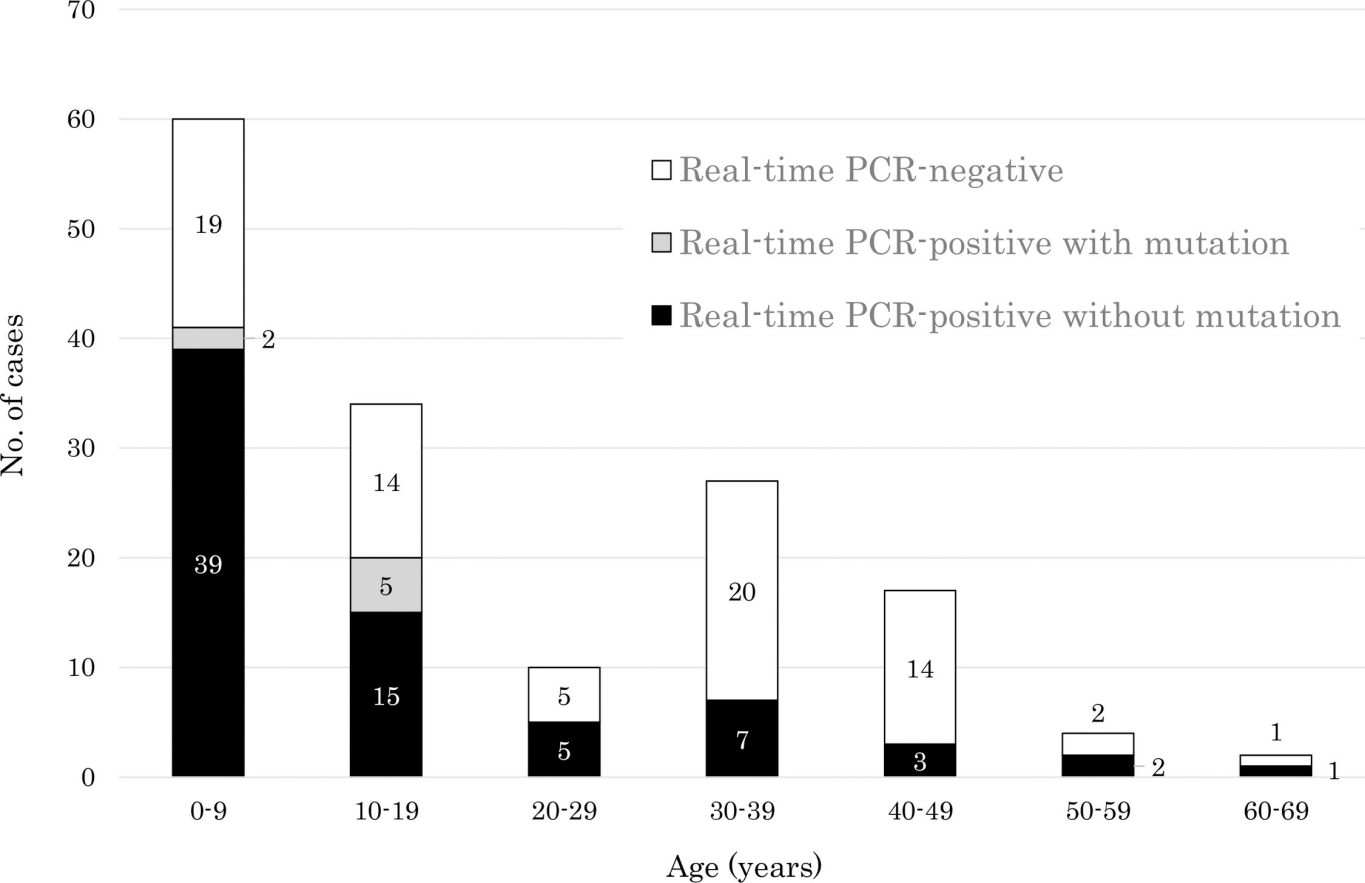
Age distribution of cases. QPCR-negative cases (white columns), qPCR-positive with a point mutation at domain V of the 23S rRNA gene of *M*. *pneumoniae* (gray columns) and qPCR-positive without a point mutation at domain V of the 23S rRNA gene of *M*. *pneumoniae* (black columns).

Pharyngeal swab samples were collected from 154 patients (64 males and 90 females) aged 11 months—68 years (average age, 19.6 years) who were diagnosed with pneumonitis (n = 112, 72.7%), bronchitis (n = 22, 14.3%), upper respiratory tract infection (n = 16, 10.4%) and others (n = 4, 2.6%). *M*. *pneumoniae* DNA was detected by qPCR from 79 (51.3%) of the 154 samples. *M*. *pneumoniae* DNA was detected from 66 (63.4%) of 104 patients under the age of 29 years and from 13 (26.0%) of 50 patients over the age of 30 years (Fig 3). Macrolide-resistant point mutations at domain V of the 23S rRNA gene were detected from 7 (8.9%) of 79 *M*. *pneumoniae* DNA-positive samples (Fig 3). Monthly enrollment (S3 Fig), period from onset of fever to pharyngeal swab sampling (S4 Fig) and clinical characteristics of the patients (S1 Table) are shown in supplements. Although statistical analysis could not be performed due to the small number of samples, the positive rate by qPCR did not differ significantly according to the period from onset of fever to sampling (S4 Fig).

### Detection of *M*. *pneumoniae* by the Smart Gene^®^ system

**Fig 4:**
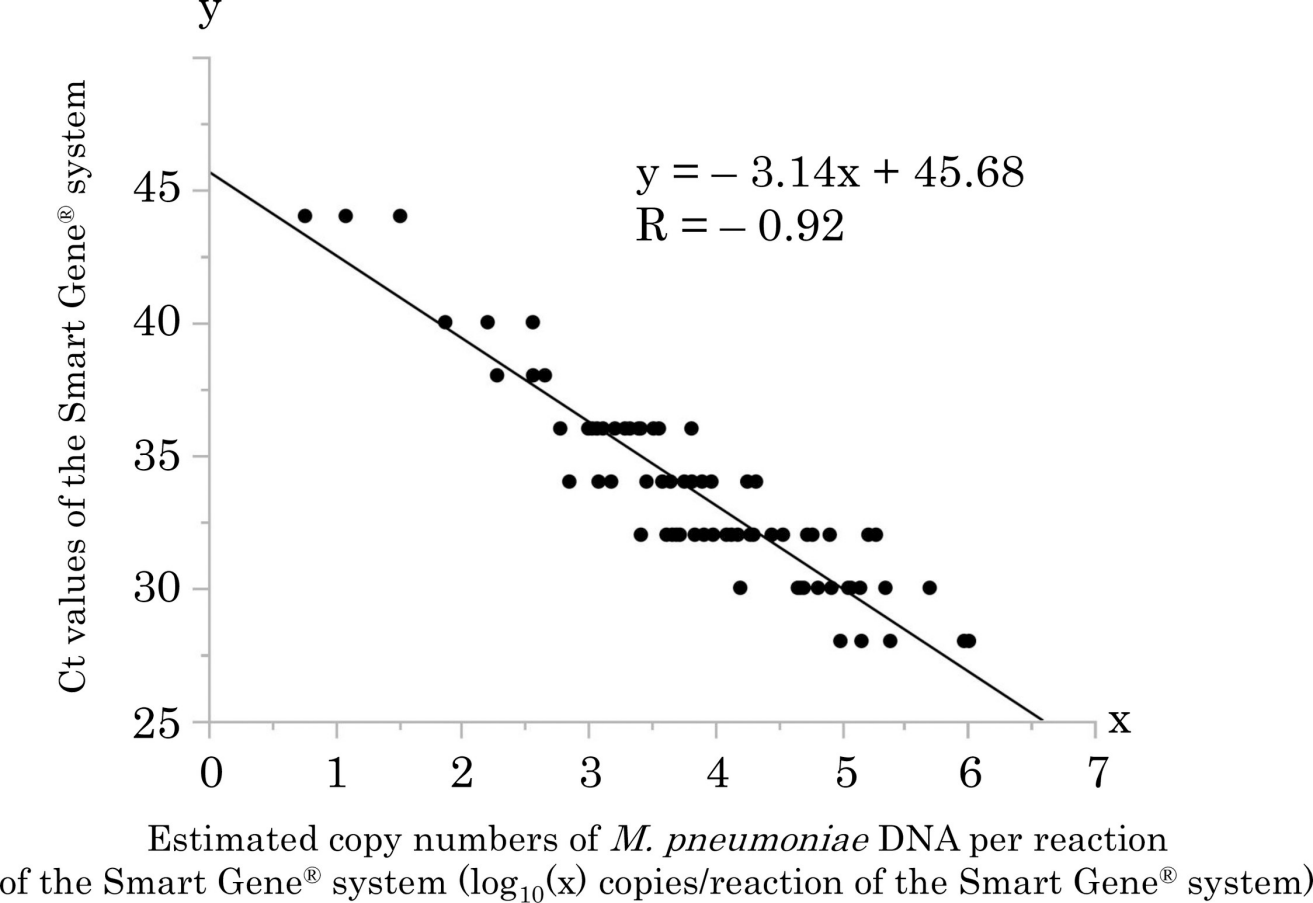
Correlation between Ct values of the Smart Gene^®^ system and copy numbers of *M*. *pneumoniae* DNA measured by qPCR. Scatter plots and fitted line showing a strong negative linear association (correlation coefficient = -0.92 (p<0.0001)) between Ct values of the Smart Gene^®^ system and estimated copy numbers of *M*. *pneumoniae* DNA per reaction of the Smart Gene^®^ system calculated from values of qPCR.

Compared with the results of qPCR, the sensitivity and specificity of the Smart Gene^®^ system were 98.7% (78/79) and 100.0% (75/75), respectively. The positive and negative predictive values of the Smart Gene^®^ system were 100.0% (78/78) and 98.7% (75/76), respectively. The diagnostic accuracy was 99.4% (153/154) (Table 1). In the sample for which the result was negative by the Smart Gene^®^ system, the estimated copy number of *M*. *pneumoniae* DNA measured by qPCR was 6.7 copies per reaction of the Smart Gene^®^ system. The threshold cycle (Ct) values of the Smart Gene^®^ system were correlated with the copy numbers of *M*. *pneumoniae* DNA measured by qPCR (correlation coefficient = -0.92, p<0.0001) (Fig 4).

**Table 1:** Sensitivity and specificity of the Smart Gene^®^ system for detection of *M*. *pneumoniae*.

		QPCR		(total)
		positive	negative	
Smart Gene^®^ system	positive	78	0	78
	negative	1	75	76
(total)		79	75	154

Sensitivity of Smart Gene^®^ system: 98.7% (78/79), 95% CI: 93.1% to 100.0%

Specificity of Smart Gene^®^ system: 100.0% (75/75), 95% CI: 95.2% to 100.0%

Positive predictive value of Smart Gene^®^ system: 100.0% (78/78), 95% CI: 95.4% to 100.0%

Negative predictive value of Smart Gene^®^ system: 98.7% (75/76), 95% CI: 92.9% to 100.0%

Diagnostic accuracy of Smart Gene^®^ system: 99.4% (153/154), 95% CI: 96.4% to 100.0%

### Detection of a point mutation at domain V of the 23S rRNA gene of *M*. *pneumoniae*

A point mutation at domain V of the 23S rRNA gene of *M*. *pneumoniae* was detected by the Smart Gene^®^ system in 7 (9.0%) of 78 samples in which *M*. *pneumoniae* was detected by the Smart Gene^®^ system. The A2063G mutation at domain V of the 23S rRNA gene was detected in 6 samples and the A2064C mutation at domain V of the 23S rRNA gene was detected in one sample by direct sequencing. In the remaining 71 *M*. *pneumoniae*-positive samples, mutations at domain V of the 23S rRNA gene were not detected (S2 Table). Therefore, the sensitivity and specificity of the Smart Gene^®^ system for detection of a point mutation at domain V of the 23S rRNA gene were 100.0% (7/7) and 100.0% (71/71), respectively. Statistical significance was observed for the differences in the prevalence of point mutations at domain V of the 23S rRNA gene between patients with and those without macrolide pre-administration: 71.4% (5 of 7) and 9.7% (7 of 72), respectively (*p* = 0.0003) (S3 Table).

### Antibiotic susceptibility

*M*. *pneumoniae* was isolated from 38 pharyngeal swab samples and *in vitro* anti-mycoplasma activities of three antibiotics (clarithromycin, minocycline and tosufloxacin) against isolates of *M*. *pneumoniae* with or without a point mutation at domain V of the 23S rRNA gene were measured (Table 2). Five strains of *M*. *pneumoniae* were isolated from samples with the A2063G mutation at domain V of the 23S rRNA gene, and 33 strains of *M*. *pneumoniae* were isolated from samples without a mutation at domain V of the 23S rRNA gene. In Japan, the most frequently administered macrolide antibiotic in 2016 was clarithromycin (3.28 DID: the defined daily dose per 1000 inhabitants per day) followed by azithromycin (0.65 DID) [38]. Clarithromycin was therefore chosen for MIC assays. The MICs of clarithromycin among the 5 isolates with the A2063G mutation at domain V of the 23S rRNA gene were >64 μg/ml, and those among the 33 isolates without a mutation at domain V of the 23S rRNA gene were <0.0625 μg/ml (Table 2). The MICs of minocycline and tosufloxacin among the 5 isolates with the A2063G mutation at domain V of the 23S rRNA gene were 2–4 and 0.5 μg/ml, respectively, and those among 33 isolates without a mutation at domain V of the 23S rRNA gene were 0.25–4 and 0.125–0.5 μg/ml, respectively (Table 2). The results for point mutations at domain V of the 23S rRNA gene of *M*. *pneumoniae* detected by the Smart Gene^®^ system were consistent with the antibiotic susceptibility of clinical isolates.

**Table 2:** Macrolide susceptibility evaluated in the Smart Gene^®^ system and MICs of antibiotics among *M*. *pneumoniae* isolates.

Case no.	Age	Sex	Macrolide susceptibility evaluated in Smart Gene^®^ system	Mutation at domain V of the 23S rRNA gene confirmed by sequencing	MIC(μg/ml)		
					CAM	MINO	TFLX
27	9	M	R	A2063G	>64	2	0.5
86	12	F	R	A2063G	>64	4	0.5
87	10	F	R	A2063G	>64	2	0.5
109	14	F	R	A2063G	>64	2	0.5
120	10	F	R	A2063G	>64	2	0.5
2	11	F	S	none	<0.0625	2	0.5
4	4	F	S	none	<0.0625	2	0.25
6	4	F	S	none	<0.0625	4	0.5
7	6	F	S	none	<0.0625	2	0.5
22	2	M	S	none	<0.0625	1	0.5
25	10	M	S	none	<0.0625	2	0.5
40	8	M	S	none	<0.0625	2	0.5
54	8	F	S	none	<0.0625	1	0.5
58	4	F	S	none	<0.0625	1	0.5
59	10	M	S	none	<0.0625	4	0.25
62	11	F	S	none	<0.0625	1	0.5
64	11	M	S	none	<0.0625	2	0.5
69	55	F	S	none	<0.0625	1	0.5
80	3	M	S	none	<0.0625	1	0.5
88	7	M	S	none	<0.0625	1	0.5
91	4	M	S	none	<0.0625	2	0.125
104	9	M	S	none	<0.0625	0.5	0.5
110	6	M	S	none	<0.0625	1	0.5
116	11	M	S	none	<0.0625	1	0.5
117	11	F	S	none	<0.0625	0.25	0.5
118	5	F	S	none	<0.0625	2	0.5
119	10	M	S	none	<0.0625	1	0.5
121	38	F	S	none	<0.0625	2	0.25
127	9	M	S	none	<0.0625	1	0.5
128	3	F	S	none	<0.0625	0.5	0.5
129	8	F	S	none	<0.0625	1	0.5
130	5	M	S	none	<0.0625	2	0.5
136	36	F	S	none	<0.0625	1	0.5
138	20	F	S	none	<0.0625	1	0.25
140	14	F	S	none	<0.0625	1	0.25
145	5	F	S	none	<0.0625	2	0.5
147	8	F	S	none	<0.0625	2	0.5
149	11	M	S	none	<0.0625	2	0.5

## Discussion

In the present study, the Smart Gene^®^ system showed sensitivity and specificity of 98.7% (78/79) and 100.0% (75/75), respectively, for the detection of *M*. *pneumoniae* from pharyngeal swab samples compared with the results of qPCR. Point mutations at domain V of the 23S rRNA gene of *M*. *pneumoniae* in 7 samples detected by the Smart Gene^®^ system were confirmed by direct sequencing. The MICs of clarithromycin among the 5 isolates with a point mutation at domain V of the 23S rRNA gene evaluated by the Smart Gene^®^ system were >64 μg/ml, and those among the 33 isolates without a mutation at domain V of the 23S rRNA gene were <0.0625 μg/ml.

Commercial molecular assays for detection of *M*. *pneumoniae* are listed in Table 3 [19, 39–49]. The major features of the Smart Gene^®^ system and other assays are summarized as follows. (1) Methods of assays: Eight assays including the Smart Gene^®^ system use qPCR and two assays use LAMP. (2) Target gene of *M*. *pneumoniae*: Two assays including the Smart Gene^®^ system use domain V of the 23S rRNA gene for detection of *M*. *pneumoniae*. The other assays use various genes (CARDS Txn, P1 adhesin, repMp4, protease-like protein and SDC1 genes). (3) Simultaneous detection of a point mutation at domain V of the 23S rRNA gene of *M*. *pneumoniae*: Two assays including the Smart Gene^®^ system concomitantly detect a point mutation at domain V of the 23S rRNA gene and the other eight assays do not detect them at the same time. (4) Inline nucleic acid extraction: Seven assays including the Smart Gene^®^ system extract nucleic acid in the inside of the instrument. In the other three assays, nucleic acid must be extracted separately. (5) Limit of detection: Detection limits of assays are displayed in various forms including colony-forming units (CFU), color changing units (CCU) and copy numbers. The limit of detection of LAMP (Eiken Chemical Co., Ltd., Japan) was reported to be 6 copies per test [50]. The multiplex Lightmix^®^ RT-PCR (TIBMolbiol, Berlin Germany) showed a limit of detection between 5 and 10 copies per test [51]. To investigate the limit of detection of the Smart Gene^®^ system, the strain *M*. *pneumoniae* MHN8-014 harboring A at the positions 2063, 2064 and 2067 (wild type) and the strain *M*. *pneumoniae* MHN8-016 harboring one transposition A2063G (mutant type) were added to the extraction reagent solution at final concentrations ranging between 1.25 and 80 copies/uL. The extraction reagent solutions containing different concentrations of *M*. *pneumoniae* were measured by the Smart Gene® system in triplicate (S5 Table). The Smart Gene® system could detect wild-type and mutant-type *M*. *pneumoniae* at a concentration of 2.5 copies/ uL in 3 of 3 measurements. In contrast, the Smart Gene® system could detect wild-type and mutant-type *M*. *pneumoniae* at a concentration of 1.25 copies/ uL in 1 of 3 measurements. In the present study, two (66.7%) of three samples for which copy numbers were under 20 copies/ uL could be detected by the Smart Gene^®^ system. Contaminants contained in clinical samples might inhibit trapping of the nucleic acids on the surface of the silica particles and might be responsible for decrease in the sensitivity. Further study using clinical samples is needed to know the limit of detection of *M*. *pneumoniae* by the Smart Gene^®^ system. (6) Size and weight of the instrument: The instruments using LAMP are small in size and light in weight, but those using qPCR are large in size and heavy in weight except for the Smart Gene^®^ system. The size of the Smart Gene^®^ system is 152 mm (W) x 343 mm (D) x 300 mm (H) and the weight is 6 kg.

**Table 3:** Comparison of commercial molecularly based assays for detection of *M*. *pneumoniae*.

	Assay (Manufacturer)	Method	Target gene	Specimen	Mutation detection	Inline nucleic acid extraction	Running time	Limit of detection	Size and weight of instrument	References
1	FilmArray RP2plus (Biofire)	QPCR	CARDS toxin	NPS	No	Yes	45min	30 CFU/mL for strain M129	254(W) × 393(D) × 165(H), 18kg	23, 24
2	ePlex RPP (GenMark)	QPCR	P1 adhesin	NPS	No	Yes	120min	300 CCU/mL for strain M129	480(W) x 540(D) x 590(H), 40kg	25
3	NxTAG RPP (Luminex)	QPCR	P1 adhesin	NPS	No	No	240min	142 CCU/mL	165(W) x 600(D) x 430(H), 18kg	14, 26, 27
4	Respiratory Bacterial ELITe MGB Panel (ELITech)	QPCR	repMp4	BAL	No	Yes	150min	0.16 CFU/test for strain M129	1000(W) x 750(D) x 850(H), 184kg	14, 28
5	Alethia Mycoplasma Direct (Meridian)	LAMP	Protease-like protein	TS	No	Yes	60min	88 CFU/test for strain FH and 7.5 CFU/test for strain M129	210(W) x 292(D) x 95(H), 3kg	29
6	QIAstat-Dx RPP (QIAGEN)	QPCR	P1 adhesin	NPS	No	Yes	120min	0.1 CFU/ml for strain M129-B7	234(W) x 517(D) x 326(H), 5kg	30
7	BioCode RPP (Applied BioCode)	QPCR	unpublished	NPS	No	No	300min	15.0 CCU/mL for strain M129	1067(W) x 788(D) x 1550(H), 68kg	31
8	Loopamp (Eiken chemical)	LAMP	SDC1	TS, NPS, S	No	No	50min	2–20 CCU/test	Control unit 190(W) x 230(D) x 106(H), 1.1kg, Amplification unit 150(W) x 275(D) x 121(H), 1.4kg	32
9	GENECUBE (Toyobo)	QPCR, QProbe	23rRNA	TS, NPS, S	Yes	Yes	30min	25 copies/test	(900(W) x 550(D) x 650(H), 92kg	33
10	Smart Gene (MIZUHO MEDY)	QPCR, QProbe	23rRNA	TS	Yes	Yes	50min	10 copies/test	152(W) x 343(D) x 300(H), 6kg	present study

Abbreviations: Loop-mediated Isothermal Amplification (LAMP), nasopharyngeal swabs (NPS), bronchoalveolar lavage (BAL), throat swabs (TS), sputum (S), expectorated sputum (ES), endotracheal aspirates (EA), color change units (CCU), colony-forming units (CFU).

The cost of the Smart Gene^®^ system is $5,000 for the gene analyzer (Smart Gene^®^) and $30 for the dedicated cartridge (Smart Gene^®^ Myco) per sample. The price of a qPCR thermal cycler ranges from $15,000 to over $90,000 and the cost of qPCR is $5 per sample. The price of equipment for LAMP (Loopamp Realtime Turbidimeter) is $18,000 and the cost of the LAMP assay is $15 per sample.

The Smart Gene^®^ system can detect macrolide resistance associated with mutations at the positions 2063, 2064 and 2067 in domain V of the 23S rRNA gene (S4 Table). However, the Smart Gene^®^ system cannot detect macrolide resistance associated with mutations at the position 2617 in domain V of the 23S rRNA gene because the specific primers of the Smart Gene^®^ system do not amplify domain V of the 23S rRNA gene containing position 2617. Because amplicons of qPCR should be ideally between 50 and 150 bp in length [52], the position 2617 at domain V of the 23S rRNA gene was not included in the amplicon of the Smart Gene^®^ system. Therefore, if patients who are suspected to be suffering from MSMP infection (positive for *M*. *pneumoniae* and negative for mutations of positions 2063, 2064 and 2067 at domain V of the 23S rRNA gene by the Smart Gene^®^) have prolonged fever or respiratory symptoms, direct sequencing might be helpful for detecting a mutation at position 2617 in domain V of the 23S rRNA gene. However, MRMP with a mutation at position 2617 in domain V of the 23S rRNA gene is rare in the world. Tanaka *et al*. reported that the most frequent mutation of MRMP was A2063G (95.8%) followed by A2063T (3.1%), A2064G (0.6%), A2063C (0.3%), C2617G (0.2%) and C2617T (0.1%) in Japan [53]. Ishiguro *et al*. reported that the most frequent mutation of MRMP was A2063G (97.5%) followed by A2064G (1.3%) and C2617T (1.3%) in Japan [54]. Diaz *et al*. reported that the most frequent mutation of MRMP was A2063G (85.7%) followed by A2064G (14.3%) in the United States [55]. Zhou *et al*. reported that all MRMP strains had A2063G (100.0%) in China [56]. Dumke *et al*. reported that all MRMP strains had A2063G (100.0%) in Germany [57]. Chironna *et al*. reported that all MRMP strains had A2063G (100.0%) in Italy [58]. Averbuch *et al*. reported that all MRMP strains had A2063G (100.0%) in Israel [59]. Furthermore, mutation at position 2617 was associated with low-level macrolide resistance compared to mutation at position 2063 or 2064 [8]. Taken together, the results indicate that the clinical impact of missing MRMP associated with a mutation at position 2617 in domain V of the 23S rRNA gene is minimal.

Recently, a dedicated cartridge of the Smart Gene^®^ system for detection of SARS-CoV-2 (Smart Gene^®^ SARS-CoV-2) has been marketed in Japan [60]. Dedicated cartridges for detection of *Clostridioides difficile*, *Helicobacter pylori*, influenza viruse*s*, *Bordetella pertussis*, *Chlamydia trachomatis* and *Neisseria gonorrhoeae* are in development. These assays will use stool, nasopharyngeal swab, nasal swab, saliva and urine as samples but will not use blood.

Limitations of this study should be recognized. Only 7 of 79 patients were infected with *M*. *pneumoniae* with a mutation in domain V of the 23S rRNA gene. This number might not be sufficient to evaluate the capacity for detection of a point mutation at domain V of the 23S rRNA gene of *M*. *pneumoniae* by the Smart Gene^®^ system. Recently, Nagita *et al*. reported that mutations in domain V of the 23S rRNA gene of *M*. *pneumoniae* were detected in 24 of 38 patients infected with *M*. *pneumoniae* using the Smart Gene^®^ system and the results were confirmed by direct sequencing, revealing all mutations as A2063G [61].

In conclusion, the present study showed high sensitivity and high specificity of the Smart Gene^®^ system for detecting *M*. *pneumoniae* from pharyngeal swab samples. The Smart Gene^®^ system can detect the existence of *M*. *pneumoniae* and a point mutation at domain V of the 23S rRNA gene of *M*. *pneumoniae* at the same time. The entire procedure of the Smart Gene^®^ system is completed within 50 min and the instrument is small (152 mm (W), 343 mm (D) and 300 mm (H)). The Smart Gene^®^ system can also be useful in countries or areas where the macrolide-resistant rate of *M*. *pneumoniae* is currently low for monitoring the trend of macrolide resistance of *M*. *pneumoniae*. Therefore, the Smart Gene^®^ system is suitable for point-of-care testing not only in a hospital setting but also in an outpatient setting.

## Supporting information

S1 Fig Detection of *M*. *pneumoniae* with the Smart Gene^®^ system. The tip of a swab containing a specimen is inserted into the extraction reagent solution vial. The tip is then squeezed while rotating it several times to extract nucleic acids (A). After placing a dropping filter on the vial (B), four drops of the extraction reagent solution containing nucleic acids are placed onto the sample spot of the cartridge (C). The cartridge is set on the insertion slot of the instrument (D).
(PPTX)

S2 Fig Nucleic acid extraction method and cartridge operation. The sample is absorbed by the absorption pad through the membrane filter, and the nucleic acids in the sample are trapped on the surface of the silica particles. NA indicates nucleic acid (A). The wash buffer tank is moved forward and the washing buffer (blue color) is released to the sample spot from the tank. The red arrowheads indicate the position of the tank (B). The membrane filter is washed by the washing buffer and the washing buffer is absorbed by the absorption pad. The support plate transfers the membrane filter containing nucleic acids into the reaction tube that contains all of the reagents necessary for QProbe PCR. The red arrowheads indicate the position of the support plate (C).
(PPTX)

S3 Fig Monthly enrollment of cases. QPCR-negative cases (white columns), qPCR-positive with a point mutation at domain V of the 23S rRNA gene of *M*. *pneumoniae* (gray columns) and qPCR-positive without a point mutation at domain V of the 23S rRNA gene of *M*. *pneumoniae* (black columns).
(PPTX)

S4 Fig Period from onset of fever to pharyngeal swab sampling. QPCR-negative cases (white columns), qPCR-positive with a point mutation at domain V of the 23S rRNA gene of *M*. *pneumoniae* (gray columns) and qPCR-positive without a point mutation at domain V of the 23S rRNA gene of *M*. *pneumoniae* (black columns).
(PPTX)

S5 Fig Antibiotic susceptibility test. Results of MIC determination using MRMP (Case no. 27) and MSPN (Case no. 2) strains after 7 days of culture. When *M*. *pneumoniae* grows, the color of the medium changes from yellow to red. Circles indicate the determined MIC.
(PPTX)

S1 Table Clinical characteristics of patients enrolled in this study. (DOCX)

S2 Table Sensitivity and specificity of the Smart Gene^®^ system for detection of a point mutation at domain V of the 23S rRNA gene of *M*. *pneumoniae*. Sensitivity of the Smart Gene^®^ system for detection of a point mutation at domain V of the 23S rRNA gene was 100.0% (7/7). Specificity of the Smart Gene^®^ system for detection of a point mutation at domain V of the 23S rRNA gene was 100.0% (71/71).
(DOCX)

S3 Table Antibiotics used before collection of specimens and point mutation at domain V of the 23S rRNA gene detected by sequencing. (DOCX)

S4 Table Detection of synthetic oligonucleotides including the sequence of domain V of the 23S rRNA gene by the Smart Gene^®^ system. Oligonucleotides of 151 nucleotides in length containing the sequence of domain V of the 23S rRNA gene of *M*. *pneumoniae* (bases 2000 to 2150 of *M*. *pneumoniae* strain M129, GenBank accession number NR_077056) were synthesized. The oligonucleotides harbor A at positions 2063, 2064 and 2067 (wild type) or harbor one of the transpositions (A2063T, A2063G, A2063C, A2064T, A2064G, A2064C and A2067G) (mutant types). The oligonucleotides were added to the extraction reagent solution to a final concentration of 40 copies/uL and measured by the Smart Gene^®^ system.
(DOCX)

S5 Table Lower limit of detection by the Smart Gene^®^ system. The strain *M*. *pneumoniae* MHN8-014 harboring A at positions 2063, 2064 and 2067 (wild type) and the strain *M*. *pneumoniae* MHN8-016 harboring one transposition A2063G (mutant type) were used. Each strain was diluted with PPLO medium and the copy numbers of *M*. *pneumoniae* in PPLO medium were measured by qPCR [13, 35, 36]. Each strain of *M*. *pneumoniae* with 80, 40, 20, 10, 5, 2.5 and 1.25 copies per one μl was prepared by twofold serial dilution using the extraction reagent solution of the Smart Gene^®^ system. The extraction reagent solutions containing different concentrations of *M*. *pneumoniae* were measured by the Smart Gene^®^ system. The measurements were done in triplicate. After measurement by the Smart Gene^®^ system, aliquots of the extraction reagent were used for confirmation of the copy number of *M*. *pneumoniae* measured by qPCR [13, 35, 36].
(DOCX)

S1 File Data set for this manuscript. (XLSX)

S2 File (DOCX)

S3 File (DOCX)
