# Supplementary figures and images for: Point-of-care molecular diagnosis of Mycoplasma pneumoniae including macrolide sensitivity using quenching probe polymerase chain reaction
Source: PLoS One. 2021 Oct 14;16(10):e0258694. doi: 10.1371/journal.pone.0258694 (PMC8516298; doi:10.1371/journal.pone.0258694)

## Slide 1
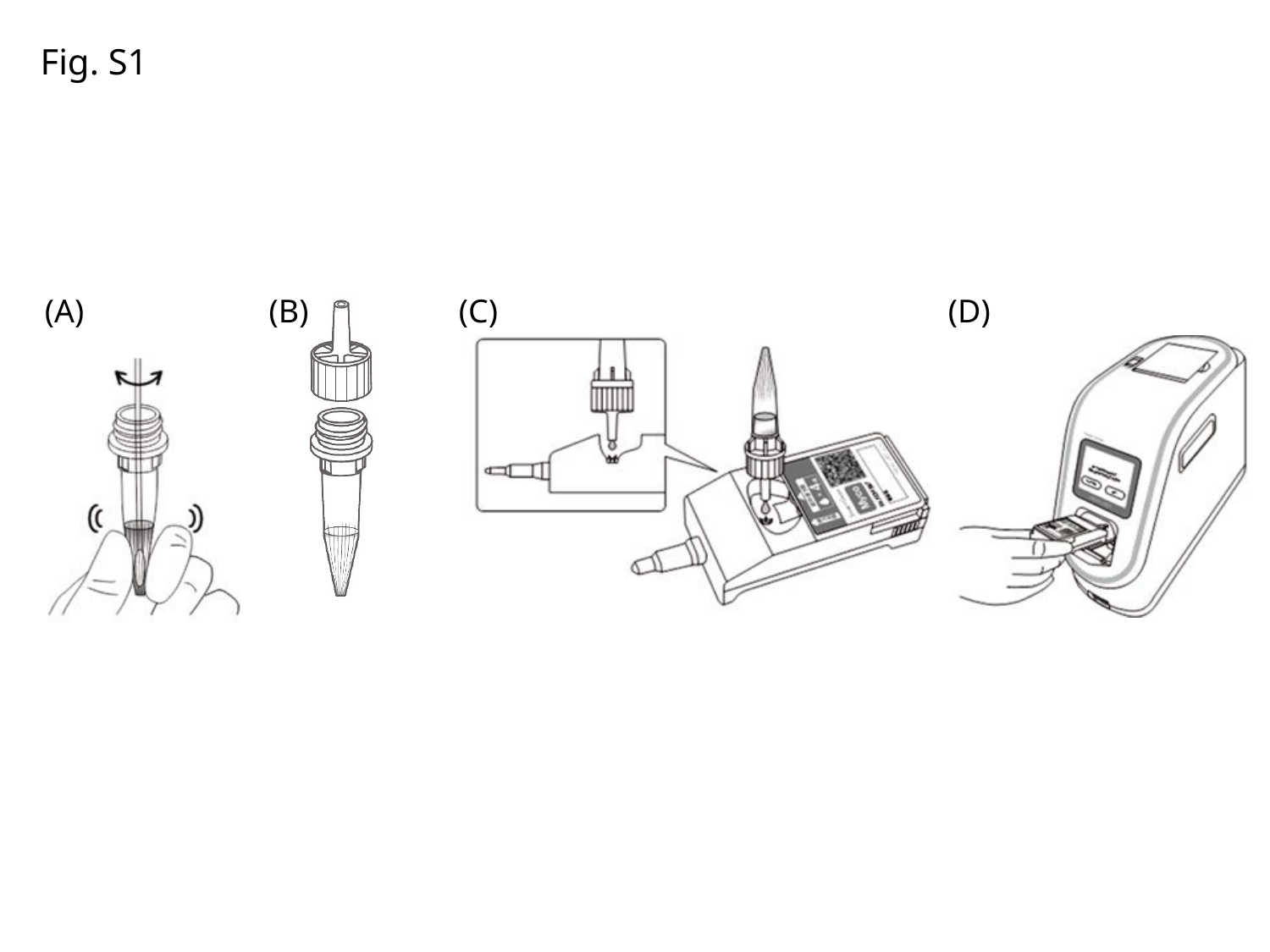

Fig. S1
(A)
(B)
(C)
(D)

Supplement: S1 Fig — The tip of a swab containing a specimen is inserted into the extraction reagent solution vial. The tip is then squeezed while rotating it several times to extract nucleic acids (A). After placing a dropping filter on the vial (B), four drops of the extraction reagent solution containing nucleic acids are placed onto the sample spot of the cartridge (C). The cartridge is set on the insertion slot of the instrument (D). (PPTX) [file pone.0258694.s001.pptx]

## Slide 1
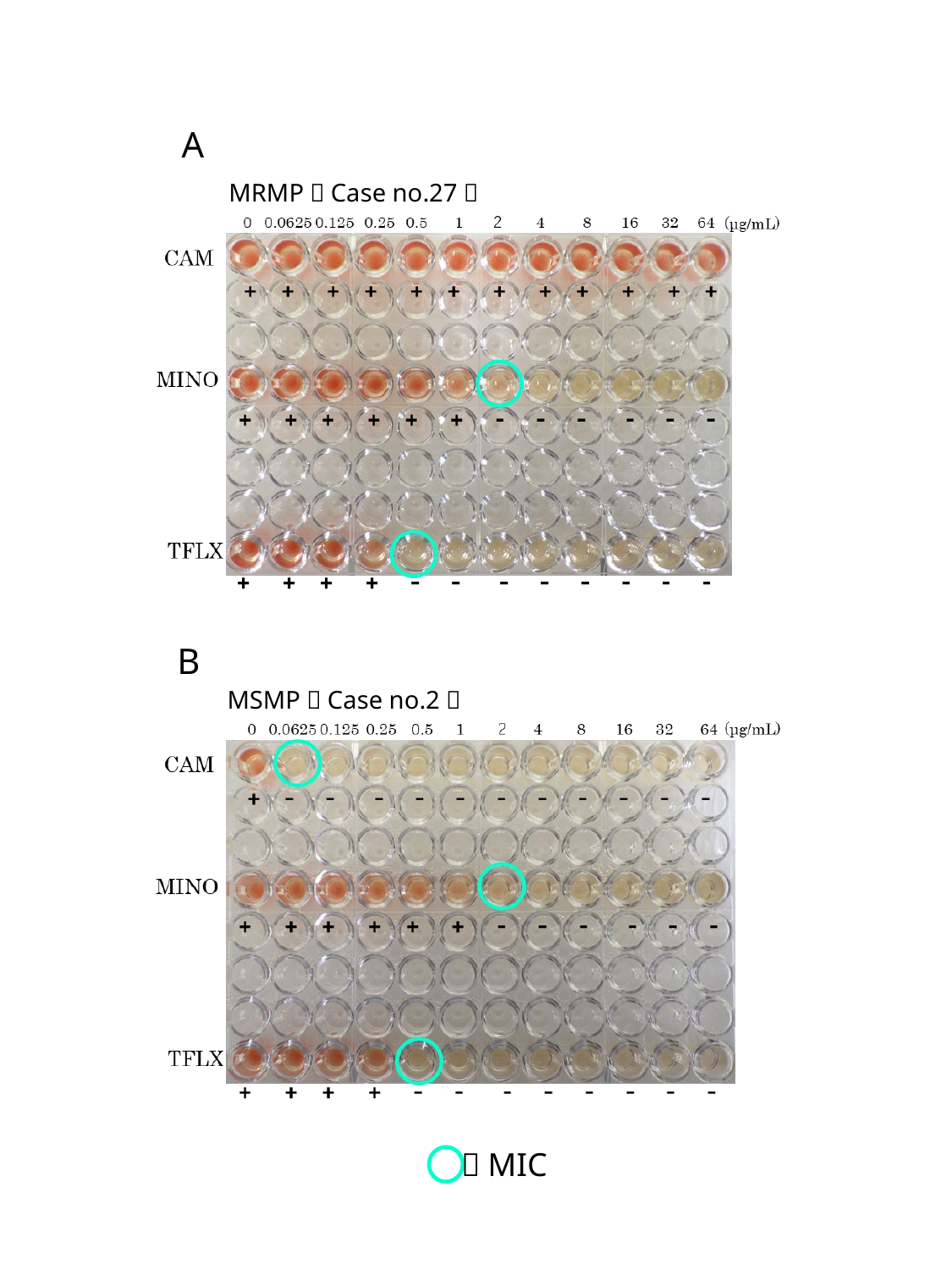

A
MRMP（Case no.27）
B
MSMP（Case no.2）
：MIC

Supplement: S5 Fig — Results of MIC determination using MRMP (Case no. 27) and MSPN (Case no. 2) strains after 7 days of culture. When M. pneumoniae grows, the color of the medium changes from yellow to red. Circles indicate the determined MIC. (PPTX) [file pone.0258694.s005.pptx]
