# Supplementary material for: Point-of-care molecular diagnosis of Mycoplasma pneumoniae including macrolide sensitivity using quenching probe polymerase chain reaction
Source: PLoS One. 2021 Oct 14;16(10):e0258694. doi: 10.1371/journal.pone.0258694 (PMC8516298; doi:10.1371/journal.pone.0258694)
Supplement: S2 Fig — The sample is absorbed by the absorption pad through the membrane filter, and the nucleic acids in the sample are trapped on the surface of the silica particles. NA indicates nucleic acid (A). The wash buffer tank is moved forward and the washing buffer (blue color) is released to the sample spot from the tank. The red arrowheads indicate the position of the tank (B). The membrane filter is washed by the washing buffer and the washing buffer is absorbed by the absorption pad. The support plate transfers the membrane filter containing nucleic acids into the reaction tube that contains all of the reagents necessary for QProbe PCR. The red arrowheads indicate the position of the support plate (C). (PPTX) [file pone.0258694.s002.pptx]

## Slide 1
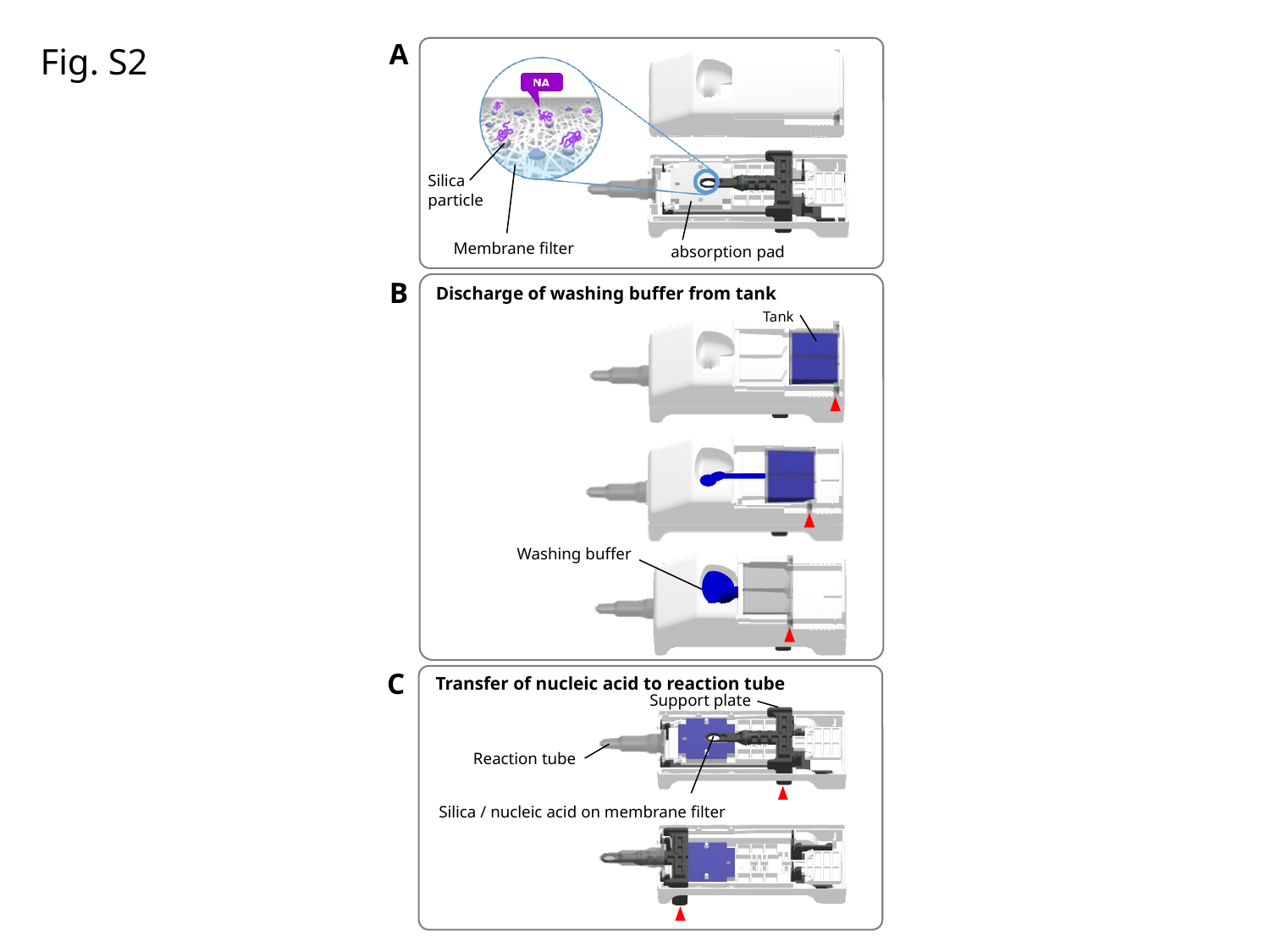

Fig. S2
A
Silica particle
Membrane filter
absorption pad
B
Discharge of washing buffer from tank
Tank
Washing buffer
C
Transfer of nucleic acid to reaction tube
Support plate
Reaction tube
Silica / nucleic acid on membrane filter
