# Supplementary material for: Point-of-care molecular diagnosis of Mycoplasma pneumoniae including macrolide sensitivity using quenching probe polymerase chain reaction
Source: PLoS One. 2021 Oct 14;16(10):e0258694. doi: 10.1371/journal.pone.0258694 (PMC8516298; doi:10.1371/journal.pone.0258694)
Supplement: S3 Fig — QPCR-negative cases (white columns), qPCR-positive with a point mutation at domain V of the 23S rRNA gene of M. pneumoniae (gray columns) and qPCR-positive without a point mutation at domain V of the 23S rRNA gene of M. pneumoniae (black columns). (PPTX) [file pone.0258694.s003.pptx]

## Slide 1
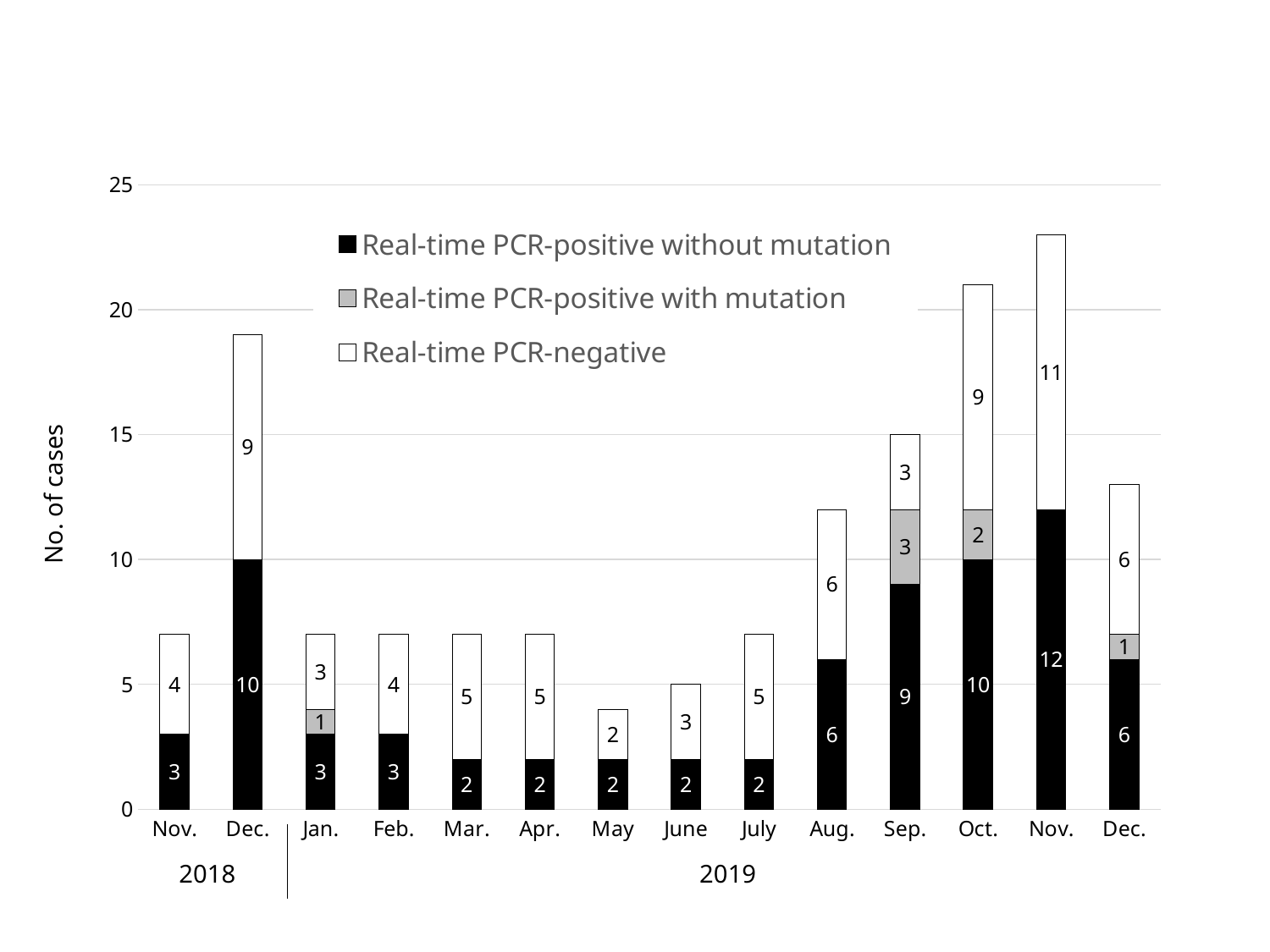

### Chart
| Category | Real-time PCR-positive without mutation | Real-time PCR-positive with mutation | Real-time PCR-negative |
|---|---|---|---|
| Nov. | 3.0 | None | 4.0 |
| Dec. | 10.0 | None | 9.0 |
| Jan. | 3.0 | 1.0 | 3.0 |
| Feb. | 3.0 | None | 4.0 |
| Mar. | 2.0 | None | 5.0 |
| Apr. | 2.0 | None | 5.0 |
| May | 2.0 | None | 2.0 |
| June | 2.0 | None | 3.0 |
| July | 2.0 | None | 5.0 |
| Aug. | 6.0 | None | 6.0 |
| Sep. | 9.0 | 3.0 | 3.0 |
| Oct. | 10.0 | 2.0 | 9.0 |
| Nov. | 12.0 | None | 11.0 |
| Dec. | 6.0 | 1.0 | 6.0 |No. of cases
2018
2019
