# Supplementary material for: Point-of-care molecular diagnosis of Mycoplasma pneumoniae including macrolide sensitivity using quenching probe polymerase chain reaction
Source: PLoS One. 2021 Oct 14;16(10):e0258694. doi: 10.1371/journal.pone.0258694 (PMC8516298; doi:10.1371/journal.pone.0258694)
Supplement: S4 Fig — QPCR-negative cases (white columns), qPCR-positive with a point mutation at domain V of the 23S rRNA gene of M. pneumoniae (gray columns) and qPCR-positive without a point mutation at domain V of the 23S rRNA gene of M. pneumoniae (black columns). (PPTX) [file pone.0258694.s004.pptx]

## Slide 1
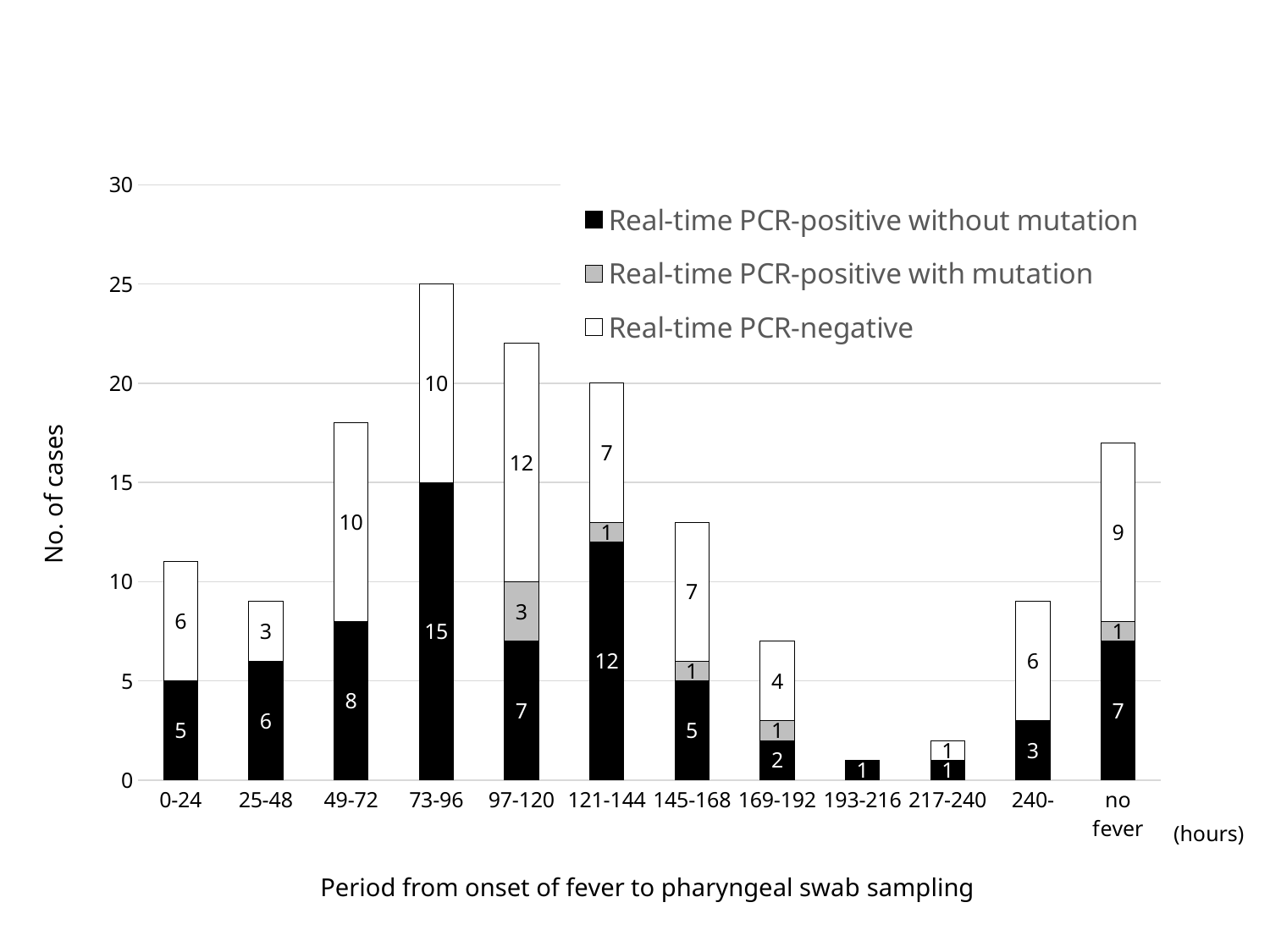

### Chart
| Category | Real-time PCR-positive without mutation | Real-time PCR-positive with mutation | Real-time PCR-negative |
|---|---|---|---|
| 0-24 | 5.0 | None | 6.0 |
| 25-48 | 6.0 | None | 3.0 |
| 49-72 | 8.0 | None | 10.0 |
| 73-96 | 15.0 | None | 10.0 |
| 97-120 | 7.0 | 3.0 | 12.0 |
| 121-144 | 12.0 | 1.0 | 7.0 |
| 145-168 | 5.0 | 1.0 | 7.0 |
| 169-192 | 2.0 | 1.0 | 4.0 |
| 193-216 | 1.0 | None | None |
| 217-240 | 1.0 | None | 1.0 |
| 240- | 3.0 | None | 6.0 |
| no fever | 7.0 | 1.0 | 9.0 |No. of cases
(hours)
Period from onset of fever to pharyngeal swab sampling
