# Supplementary material for: Point-of-care molecular diagnosis of Mycoplasma pneumoniae including macrolide sensitivity using quenching probe polymerase chain reaction
Source: PLoS One. 2021 Oct 14;16(10):e0258694. doi: 10.1371/journal.pone.0258694 (PMC8516298; doi:10.1371/journal.pone.0258694)
Supplement: S1 Table — (DOCX) [file pone.0258694.s006.docx]

S1 Table. Clinical characteristics of the patients enrolled in this study

|  | Total (n=154) | Real-time PCR-positive (%) (n=79) | Real-time PCR-negative (%) (n=75) | *P* value |
| --- | --- | --- | --- | --- |
| Age (year) |  |  |  |  |
| Mean ± SD | 19.6 ± 16.5 | 14.9 ± 14.7 | 24.7 ± 16.8 | 0.001* |
| Range | 0 – 68 | 2 – 68 | 0 – 63 |  |
| No. (%) females | 90 (58.4%) | 46 (51.1%) | 44 (48.9%) | 1.000 |
| No. (%) males | 64 (41.6%) | 33 (51.6%) | 31 (48.4%) |  |
| Signs & symptoms |  |  |  |  |
| Fever (37.5˚C) | 137 (89.0%) | 71 (89.9%) | 66 (88.0%) | 0.800 |
| Cough | 149 (96.8%) | 78 (98.7%) | 71 (94.7%) | 0.201 |
| Nasal discharge | 44 (28.6%) | 15 (19.0%) | 29 (38.7%) | 0.008* |
| Sore throat | 34 (22.1%) | 15 (19.0%) | 19 (25.3%) | 0.437 |
| Wheezing | 10 (6.5%) | 6 (7.6%) | 4 (5.3%) | 0.748 |
| Dyspnea | 28 (18.2%) | 11 (13.9%) | 17 (22.7%) | 0.210 |
| Headache | 30 (19.5%) | 13 (16.5%) | 17 (22.7%) | 0.416 |
| Fatigue | 21 (13.7%) | 10 (12.7%) | 11 (14.9%) | 0.816 |
| Others | 19 (12.3%) | 7 (8.9%) | 12 (16.0%) | 0.223 |
| Diagnosis |  |  |  |  |
| Pneumonitis | 112 (72.7%) | 64 (81.0%) | 48 (64.0%) | 0.020 |
| Bronchitis | 22 (14.3%) | 9 (11.4%) | 13 (17.3%) | 0.359 |
| Upper respiratory infection | 16 (10.4%) | 6 (7.6%) | 10 (13.3%) | 0.296 |
| Others | 4 (2.6%) | 0 (0.0%) | 4 (5.3%) | 0.125 |
