# Supplementary material for: Point-of-care molecular diagnosis of Mycoplasma pneumoniae including macrolide sensitivity using quenching probe polymerase chain reaction
Source: PLoS One. 2021 Oct 14;16(10):e0258694. doi: 10.1371/journal.pone.0258694 (PMC8516298; doi:10.1371/journal.pone.0258694)
Supplement: S2 Table — Sensitivity of the Smart Gene® system for detection of a point mutation at domain V of the 23S rRNA gene was 100.0% (7/7). Specificity of the Smart Gene® system for detection of a point mutation at domain V of the 23S rRNA gene was 100.0% (71/71). (DOCX) [file pone.0258694.s007.docx]

S2 Table. Sensitivity and specificity of the Smart Gene^®^ system for detection of a point mutation at domain V of the 23S rRNA gene of *M. pneumoniae*

|  | | Direct sequencing | | (total) |
| --- | --- | --- | --- | --- |
|  |  | mutation-positive | mutation-negative |  |
| Smart Gene^®^ system | mutation-positive | 7 | 0 | 7 |
|  | mutation-negative | 0 | 71 | 71 |
| (total) | | 7 | 71 | 78 |

Sensitivity of the Smart Gene^®^ system for detection of a point mutation at domain V of the 23S rRNA gene: 100.0% (7/7)

Specificity of the Smart Gene^®^ system for detection of a point mutation at domain V of the 23S rRNA gene: 100.0% (71/71)
