# Supplementary material for: Point-of-care molecular diagnosis of Mycoplasma pneumoniae including macrolide sensitivity using quenching probe polymerase chain reaction
Source: PLoS One. 2021 Oct 14;16(10):e0258694. doi: 10.1371/journal.pone.0258694 (PMC8516298; doi:10.1371/journal.pone.0258694)
Supplement: S3 Table — (DOCX) [file pone.0258694.s008.docx]

S3 Table. Antibiotics used before collection of specimens and point mutation at domain V of the 23S rRNA gene detected by sequencing

| Antibiotics used before collection of specimens | Point mutation at domain V of the 23S rRNA gene detected by sequencing | | *P* value |
| --- | --- | --- | --- |
|  | With mutation (n=7) | Without mutation (n=72) |  |
| Macrolides | 5^#, †^ (71.4%) | 7^§^ (9.7%) | 0.001* |
| Quinolones | 0 (0.0%) | 1 (1.4%) | 1.000 |
| Tetracyclines | 1^†^ (14.3%) | 0 (0.0%) | 0.089 |
| β-lactam antibiotics | 1^#^ (14.3%) | 18^§^ (25.0%) | 1.000 |
| No antibiotics | 2 (28.6%) | 48 (66.7%) | 0.093 |

^#^ One patient took macrolide and β-lactam antibiotics. ^†^One patient took macrolide and tetracycline. ^§^ Two patients took macrolide and β-lactam antibiotics.
