# Supplementary material for: Point-of-care molecular diagnosis of Mycoplasma pneumoniae including macrolide sensitivity using quenching probe polymerase chain reaction
Source: PLoS One. 2021 Oct 14;16(10):e0258694. doi: 10.1371/journal.pone.0258694 (PMC8516298; doi:10.1371/journal.pone.0258694)
Supplement: S4 Table — Oligonucleotides of 151 nucleotides in length containing the sequence of domain V of the 23S rRNA gene of M. pneumoniae (bases 2000 to 2150 of M. pneumoniae strain M129, GenBank accession number NR_077056) were synthesized. The oligonucleotides harbor A at positions 2063, 2064 and 2067 (wild type) or harbor one of the transpositions (A2063T, A2063G, A2063C, A2064T, A2064G, A2064C and A2067G) (mutant types). The oligonucleotides were added to the extraction reagent solution to a final concentration of 40 copies/uL and measured by the Smart Gene® system. (DOCX) [file pone.0258694.s009.docx]

S4 Table. Detection of synthetic oligonucleotides including the sequence of domain V of the 23S rRNA gene by the Smart Gene^®^ system

| Mutation in synthetic oligonucleotides | Results of the Smart Gene^®^ system | |
| --- | --- | --- |
|  | Presence of oligonucleotides | Presence of mutation |
| Wild type | Detected | Mutation negative |
| A2063T | Detected | Mutation positive |
| A2063G | Detected | Mutation positive |
| A2063C | Detected | Mutation positive |
| A2064T | Detected | Mutation positive |
| A2064G | Detected | Mutation positive |
| A2064C | Detected | Mutation positive |
| A2067G | Detected | Mutation positive |

Oligonucleotides of 151 nucleotides in length containing the sequence of domain V of the 23S rRNA gene of *M. pneumoniae* (bases 2000 to 2150 of *M. pneumoniae* strain M129, GenBank accession number NR_077056) were synthesized. The oligonucleotides harbor A at positions 2063, 2064 and 2067 (wild type) or harbor one of the transpositions (A2063T, A2063G, A2063C, A2064T, A2064G, A2064C and A2067G) (mutant types). The oligonucleotides were added to the extraction reagent solution to a final concentration of 40 copies/uL and measured by the Smart Gene^®^ system.
