# Supplementary material for: Point-of-care molecular diagnosis of Mycoplasma pneumoniae including macrolide sensitivity using quenching probe polymerase chain reaction
Source: PLoS One. 2021 Oct 14;16(10):e0258694. doi: 10.1371/journal.pone.0258694 (PMC8516298; doi:10.1371/journal.pone.0258694)
Supplement: S5 Table — The strain M. pneumoniae MHN8-014 harboring A at positions 2063, 2064 and 2067 (wild type) and the strain M. pneumoniae MHN8-016 harboring one transposition A2063G (mutant type) were used. Each strain was diluted with PPLO medium and the copy numbers of M. pneumoniae in PPLO medium were measured by qPCR [13, 35, 36]. Each strain of M. pneumoniae with 80, 40, 20, 10, 5, 2.5 and 1.25 copies per one μl was prepared by twofold serial dilution using the extraction reagent solution of the Smart Gene® system. The extraction reagent solutions containing different concentrations of M. pneumoniae were measured by the Smart Gene® system. The measurements were done in triplicate. After measurement by the Smart Gene® system, aliquots of the extraction reagent were used for confirmation of the copy number of M. pneumoniae measured by qPCR [13, 35, 36]. (DOCX) [file pone.0258694.s010.docx]

S5 Table. Lower limit of detection by the Smart Gene^®^ system

| Strain | Copy numbers of *M. pneumoniae* in one μl of extraction reagent | |  | Results of the Smart Gene^®^ system | | | |
| --- | --- | --- | --- | --- | --- | --- | --- |
|  | Theoretical value | Observed value |  | Detection of *M. pneumoniae* | Mutation | Ct value | Average |
| MHN8-014 | 80 | 72.53 |  | + | No | 38 |  |
|  |  |  |  | + | No | 40 | 39.33 |
|  |  |  |  | + | No | 40 |  |
|  | 40 | 36.26 |  | + | No | 40 |  |
|  |  |  |  | + | No | 42 | 41.33 |
|  |  |  |  | + | No | 42 |  |
|  | 20 | 18.13 |  | + | No | 42 |  |
|  |  |  |  | + | No | 42 | 42.00 |
|  |  |  |  | + | No | 42 |  |
|  | 10 | 9.07 |  | + | No | 44 |  |
|  |  |  |  | + | No | 44 | 43.33 |
|  |  |  |  | + | No | 42 |  |
|  | 5 | 4.53 |  | + | No | 46 |  |
|  |  |  |  | + | No | 44 | 44.67 |
|  |  |  |  | + | No | 44 |  |
|  | 2.5 | 2.27 |  | + | No | 46 |  |
|  |  |  |  | + | No | 46 | 45.33 |
|  |  |  |  | + | No | 44 |  |
|  | 1.25 | 1.13 |  | + | No | 46 |  |
|  |  |  |  | - | - | - | 46.00 |
|  |  |  |  | - | - | - |  |
| MHN8-016 | 80 | 92.10 |  | + | Yes | 40 |  |
|  |  |  |  | + | Yes | 38 | 39.33 |
|  |  |  |  | + | Yes | 40 |  |
|  | 40 | 46.05 |  | + | Yes | 40 |  |
|  |  |  |  | + | Yes | 42 | 40.67 |
|  |  |  |  | + | Yes | 40 |  |
|  | 20 | 23.03 |  | + | Yes | 42 |  |
|  |  |  |  | + | Yes | 42 | 42.00 |
|  |  |  |  | + | Yes | 42 |  |
|  | 10 | 11.51 |  | + | Yes | 44 |  |
|  |  |  |  | + | Yes | 44 | 43.33 |
|  |  |  |  | + | Yes | 42 |  |
|  | 5 | 5.76 |  | + | Yes | 44 |  |
|  |  |  |  | + | Yes | 44 | 44.00 |
|  |  |  |  | + | Yes | 44 |  |
|  | 2.5 | 2.88 |  | + | Yes | 46 |  |
|  |  |  |  | + | Yes | 42 | 44.00 |
|  |  |  |  | + | Yes | 44 |  |
|  | 1.25 | 1.44 |  | + | Yes | 44 |  |
|  |  |  |  | - | - | - | 44.00 |
|  |  |  |  | - | - | - |  |
