# Supplementary material for: Point-of-care molecular diagnosis of Mycoplasma pneumoniae including macrolide sensitivity using quenching probe polymerase chain reaction
Source: PLoS One. 2021 Oct 14;16(10):e0258694. doi: 10.1371/journal.pone.0258694 (PMC8516298; doi:10.1371/journal.pone.0258694)
Supplement: S2 File — (DOCX) [file pone.0258694.s012.docx]

**Title Direct sequencing**

**ABSTRACT** This protocol shows the direct sequencing procedure to investigate the presence of mutations in 2063, 2064, and 2067 of the *M. pneumoniae* 23S rRNA gene.

**MATERIALS**

Premix Taq Catalog # R004A Takara Bio Inc.

MiniElute PCR purification kit

Catalog # 28006 QIAGEN N.V.

BigDye Terminator v3.1 Cycle Sequencing Kit

Catalog # 10977015 Thermo Fisher Scientific K.K.

UltraPureTM Dnase/Rnase-Free Distilled Water

Catalog # 4337457 Thermo Fisher Scientific K.K.

SafeSeal Micro Tube, 1.5ml

Catalog # 72.706.201 Sarstedt K.K.

All oligo sequences are listed below:

Oligo Vendor Grade concentration sequence

MN23SDVF ngrl HPLC 50μM GCAGTGAAGAACGAGGGG

MN23SDVR ngrl HPLC 50μM GTCCTCGCTTCGGTCCTCTCG

MCR seqF1 ngrl HPLC 50μM AACTATAACGGTCCTAAGGTAGCG

MCR seqR1 ngrl HPLC 50μM GCTCCTACCTATTCTCTACATGAT

Source:

Antimicrob. Agents Chemother. 2004; 48: 4624-30.

Antimicrob Agents Chemother. 2008; 52: 3542-9.

**Procedure**

1. Prepare amplification mix for the 1st PCR as follows:

2×TaKaRa Premix Taq 25μl

MN23SDVF 0.6μl

MN23SDVR 0.6μl

DDW 21.8μl

Total 48.0μl

1. Add 2μl of DNA solution to 48.0μl of amplification mix for the 1st PCR.
2. Measure with the following temperature profiles using a thermal cycler:

Step Temperature Time Cycles

Initial Denaturation 94°C 120sec ×1

Denaturation 94°C 45sec ×35

Annealing 55°C 60sec

Extension 72°C 72sec

Final extension 72°C 5min

1. Prepare amplification mix for 2nd PCR as follows:

2×TaKaRa Premix Taq 25μl

MCR seqF1 0.2μl

MCR seqR1 0.2μl

DDW 23.6μl

Total 49.0μl

1. Add 1μl of the first PCR product diluted 50-fold with DDW to 49.0μl of amplification mix for the 2nd PCR.
2. Measure with the following temperature profiles using a thermal cycler:

Step Temperature Time Cycles

Initial Denaturation 95°C 120sec ×1

Denaturation 95°C 15sec ×30

Annealing 60°C 30sec

Extension 72°C 60sec

1. Purify DNA from 50 μl of PCR reaction solution using a MiniElute PCR purification kit. Elution is performed in 15μl Buffer EB.
2. Prepare the sequencing reactions by adding MCR seqF1 primer and purified PCR product using a BigDye Terminator v3.1 Cycle Sequencing Kit and perform sequencing (3730xl DNA Analyzer, Applied Biosystems).
3. Compare with the sequence of M129 strain (Acc no. CP017343) using BioEdit (DNA Sequencing Software).
