# Supplementary material for: Point-of-care molecular diagnosis of Mycoplasma pneumoniae including macrolide sensitivity using quenching probe polymerase chain reaction
Source: PLoS One. 2021 Oct 14;16(10):e0258694. doi: 10.1371/journal.pone.0258694 (PMC8516298; doi:10.1371/journal.pone.0258694)
Supplement: S3 File — (DOCX) [file pone.0258694.s013.docx]

**Title qPCR**

**ABSTRACT** This protocol shows the DNA purification procedure using the SMITEST EX-R&D DNA extraction kit and the qPCR procedure for *M. pneumoniae* CARDS toxin gene using the purified DNA.

**MATERIALS**

SMITEST EX-R&D Catalog # GS-J0201 Medical & Biological Laboratories Co.

Isopropyl alcohol Catalog # 000-64783 KISHIDA CHEMICAL Co., Ltd.

Ethanol (99.5) Catalog # 000-28555 KISHIDA CHEMICAL Co., Ltd.

TE Buffer Catalog # 12090015 Thermo Fisher Scientific K.K.

TaqMan Gene Expression Master Mix

Catalog # 4369016 Thermo Fisher Scientific K.K.

UltraPureTM Dnase/Rnase-Free Distilled Water

Catalog # 10977015 Thermo Fisher Scientific K.K.

SafeSeal Micro Tube, 1.5ml

Catalog # 72.706.201 Sarstedt K.K.

All oligo sequences are listed below:

Oligo Vendor Grade concentration sequence

Mp181-F ngrl HPLC 50μM TTTGGTAGCTGGTTACGGGAAT

Mp181-R ngrl HPLC 50μM GGTCGGCACGAATTTCATATAAG

Mp181-P Eurofins Genomics KK

HPLC 10μM [FAM]-TGTACCAGAGCACCCCAGAAGGGCT-[BHQ-1]

Source: J. Clin. Microbiol. 2008, 46(9):3116.

Gene target: CARDS toxin (GenBank accession no. DQ447750)

Assay product: 73bp

Standard plasmid: pT7Blue with the target sequence of M129 strain in insert (Novagene, Madison, WI, USA)

**Procedure for nucleic acid extraction using a SMITEST EX-R&D DNA extraction kit**

1. Dispense 15μl of SMITEST EX-R&D Solution I into a 1.5-ml tube.
2. Add 380μl of Solution II.
3. Add 5μl of Solution IV and mix.
4. Add 100μl of the sample.
5. After mixing with a vortex mixer, incubate at 55°C for 30 minutes.
6. After spin down, add 250 μl of protein dissolvent (Solution III), mix, and incubate at 55°C for 15 minutes.
7. After spin down, add 600 μl of isopropanol, mix thoroughly by inverting, and cool on ice for at least 15 minutes.
8. Centrifuge at 12,000xg for 10 minutes at 4°C.
9. Remove supernatant with a pipette.
10. Add 500 μl of 70% ethanol, mix gently, and centrifuge at 12,000xg for 3 minutes at 4°C.
11. Remove supernatant with a pipette.
12. Add 500 μl of 70% ethanol, mix gently, and centrifuge at 12,000xg for 3 minutes at 4°C.
13. Remove supernatant with a pipette.
14. Dry obtained pellets under reduced pressure (2-3 minutes).
15. Redissolve in 15 μl of TE Buffer.
16. Leave for 15 minutes at 37°C.

**Procedure for qPCR using TaqMan Gene Expression Master Mix**

1. Prepare amplification mix as follows:

TaqMan Gene Expression Master Mix 10μl

Mp181-F 0.2μl

Mp181-R 0.2μl

Mp181-P 0.2μl

DDW 8.4μl

Total 19.0μl

1. Add 1 μl of DNA solution purified with a SMITEST EX-R&D DNA extraction kit to 19.0 μl of amplification mix.
2. Prepare calibration curve sample by diluting STANDARD plasmid with TE buffer to final concentrations of 1 copy/μl, 10 copies/μl, 100 copies/μl, and 1000 copies/μl and add 1 μl to 19.0 μl of amplification mix.
3. Measure with the following temperature profiles using a thermal cycler:

Step Temperature Time Cycles

UDG Incubation 50°C 2min ×1

Initial Denaturation 95°C 10min ×1

Denaturation 95°C 15sec ×45

Annealing/Extension 60°C 1min
